# Phenotypic and Molecular Characterization of Clinical Isolates of Vancomycin-Resistant *Enterococcus faecium* in the Health District of Bolzano (Italy) During 2021–2023

**DOI:** 10.3390/pathogens15020143

**Published:** 2026-01-28

**Authors:** Angela Maria Di Pierro, Richard Aschbacher, Maria Del Grosso, Monica Monaco, Elisabetta Pagani

**Affiliations:** 1 Laboratory of Microbiology and Virology, Provincial Hospital of Bolzano (SABES-ASDAA), Teaching Hospital of Paracelsus Medical University (PMU), 39100 Bolzano-Bozen, Italy; richard.aschbacher@sabes.it (R.A.); elisabetta.pagani@sabes.it (E.P.); 2 Department of Infectious Diseases, Istituto Superiore di Sanità, 00161 Rome, Italy; maria.delgrosso@iss.it (M.D.G.); monica.monaco@iss.it (M.M.)

**Keywords:** VanA, VanB, sequence type, NGS, antimicrobial resistance genes

## Abstract

Vancomycin-resistant *Enterococcus faecium* (VREfm) is an emerging pathogen responsible for healthcare-associated infections. For this reason, 44 VREfm isolates collected during 2021–2023 were characterized using phenotypic and genomic approaches. VREfm isolates were identified by MALDI-TOF and antimicrobial susceptibility tests were performed with Vitek 2, Sensititre, or E-test. Sequence type (ST), antibiotic resistance genes, virulence factors and genetic relatedness were determined using Next Generation Sequencing. Forty-three isolates had a VanA phenotype and *vanHAX* genotype and one had a VanB phenotype and *vanHBX* genotype. Isolates showed high antibiotic resistance to various antibiotics, but generally remained susceptible to quinupristin/dalfopristin, tigecycline and eravacycline. Two isolates were resistant to linezolid, showing the chromosomal mutation G2576T in domain V of the 23S rRNA gene in one isolate, and the transferable linezolid resistance genes *cfr(D)* and *optrA* in the other. Thirty-eight isolates belonged to ST80, one to ST17 (ST80 and ST17 are included in CC17) and one to ST697. Genomic analysis of the ST80 isolates showed that nearly all of them belonged to a single cluster. To prevent further spread of VREfm in the nosocomial environment, in addition to the application of up-to-date infection control strategies and antibiotic stewardship programs, the implementation of genomic surveillance is recommended.

## 1. Introduction

Vancomycin-resistant enterococci (VREs), mainly represented by *Enterococcus faecium*, are emerging pathogens responsible for a significant number of healthcare-associated infections. Even though most infections originate from the patient’s endogenous flora, VREs can spread from person to person by direct or indirect contact, such as through contaminated environmental surfaces or via caregivers’ hands. VREs can cause hospital outbreaks that can have significant impact on mortality, morbidity and length of patient stay, resulting in a significant financial burden on healthcare systems [1,2].

In particular, the rapid and continuous increase in bloodstream infections caused by vancomycin-resistant *E. faecium* (VREfm) in the European Union/European Economic Area, including Italy, is a cause for serious concern [3,4]. Consistent with national data, there has been a significant and gradual increase in the occurrence of VREfm infections in recent years up to 2022 in the Autonomous Province of Bolzano, located in Northern Italy, especially in the Bolzano Health District, with a reversing trend in 2023. Specifically, in the Autonomous Province of Bolzano, the percentage of VREfm isolated from blood and cerebrospinal fluid was 24.4% (10/41) in 2022, whereas in 2023 it decreased to 16.7% (6/36); all of these isolates had the VanA phenotype (defined as resistance to vancomycin and teicoplanin) [5].

This study shows phenotypic and genomic characteristics of 44 clinical VREfm isolates, 43 of them obtained from patients admitted to the Bolzano Regional Hospital during 2021–2023, together with one isolate from the Brunico Health District, also located in the Province of Bolzano. Microbial typing through Whole Genome Sequencing (WGS) was used to determine the antibiotic resistance and virulence gene content of the isolates and their possible clonal relationship.

## 2. Materials and Methods

### 2.1. Collection and Phenotypic Characterization of E. faecium VRE Isolates

Forty-four VREfm isolates (first isolate from each patient) were obtained during 2021–2023 from routine clinical specimens sent to the Clinical Microbiology Laboratory at the Bolzano Regional Hospital for diagnostic investigations of patients with suspected infections or from rectal surveillance screening. VREfm routine clinical isolates were grown on Columbia Agar and Columbia CNA Agar plates, both with sheep blood (Thermo Fisher Scientific, Waltham, MA, USA), whereas rectal surveillance isolates were grown on Brilliance VRE Agar plates (Thermo Fisher Scientific, Waltham, MA, USA), incubated for 24 h at 37 °C under aerobic conditions, and presumptive VREfm colonies have been identified by their characteristic morphology.

Identification of the isolates was performed using Matrix-assisted laser desorption/ionization-time of flight (MALDI-TOF) mass spectrometry (Bruker Daltonics MALDI Biotyper Sirius, Bremen, Germany) and antimicrobial susceptibility was determined by Vitek 2 AST-P658 cards. The isolates were subsequently stored at −80 °C using the CRYOBEADS system (bioMérieux, Marcy l’Etoile, France).

Three different systems were used for the determination of the antibiotic susceptibility of the stored isolates, in accordance with manufacturer’s specifications: Vitek 2 AST-P658 cards (bioMérieux, Marcy l’Etoile, France), Sensititre™ Streptococcus FDANDSF plates (Thermo Fisher Scientific, Waltham, MA, USA) or E-test strips (bioMérieux, Marcy l’Etoile, France); this allowed to test an expanded panel of antibiotics and the confirmation of susceptibility results between methods. The reference strains *Staphylococcus aureus* ATCC 29213, *E. faecalis* ATCC 29212 and VanB-*E. faecalis* ATCC 51299 were used for quality control.

Minimal inhibitory concentrations (MICs) were interpreted following the European Committee on Antimicrobial Susceptibility Testing breakpoints (EUCAST—version 13.0, 2023) [6]. Where clinical breakpoints were absent, EUCAST epidemiological cut-off (ECOFF) values were applied, and where neither clinical interpretation nor ECOFF values were available, MIC values alone are reported, because even without breakpoints MICs can provide comparable and meaningful data that can support clinical judgment, surveillance and future interpretation.

### 2.2. VREfm Genomic Characterization and Analysis

Single colonies of each *E. faecium* isolate, grown overnight on Columbia blood agar plates (Thermo Fisher Scientific, Waltham, MA, USA), were resuspended in 180 µL of enzyme solution (20 mg/mL Lysozyme, Tris HCl 20 nM, EDTA 2 mM, Triton 1.2%) and incubated for 30 min at 37 °C. Genomic DNA (gDNA) extraction was performed using the QIAmp^®^DNA Blood Mini Kit (QIAGEN Inc. GmbH, Hilden, Germany) according to the protocol provided by the manufacturer for Gram-positive bacteria; the quality of the extracted DNA was assessed with an automated electrophoresis system (Agilent Tape Station 4150, Santa Clara, CA, USA). Final samples were eluted in 50 µL of elution buffer and stored in the freezer at −20 °C.

Genomic libraries were prepared using the Nextera™ XT DNA Library Preparation Kit (Illumina, San Diego, CA, USA). The final concentration of the libraries was determined with the Qubit 4 fluorometer (ThermoFisher Scientific, Waltham, MA, USA), and their quality was assessed with the automated TapeStation 4150 platform (Agilent, Santa Clara, CA, USA). Sequencing of the bacterial genome was performed on the Miseq™ platform (Illumina). The quality of raw sequence data (Fastq) was assessed with the FastQC program (version 1.0.0, BaseSpace Labs); the trimming of the reads was performed with Trimmomatic (Galaxy Version 0.38.1) [7] and SPAdes (Galaxy Version 3.14.1+galaxy1) was employed for assembly, following the default settings of the server [8]. The quality of assembled genomes was assessed through the QUAST (Quality Assessment Tool for Genome Assemblies) program (Galaxy Version 5.0.2+galaxy1) [9]. Assembled genomes were analyzed by using tools available at the Center for Genomic Epidemiology (CGE-Technical University of Denmark) [10]: the identity of sequenced isolates was confirmed by KmerFinder (version 3.2); Multi Locus Sequencing Type (MLST) identification was performed by MLST (version 2.0.9), resistance determinants were searched by ResFinder (version 4.1), the LRE-Finder (version 1.0) was used for the identification of genes and mutations determining linezolid resistance and plasmid replicons were identified using PlasmidFinder (version 2.1). In addition, the AMRFinderPlus tool (Galaxy Version 1.0.19+galaxy1), developed by the National Center for Biotechnology Information (NCBI), was used for identifying point mutations. The presence of virulence factor (VF) determinants was analyzed by VFanalyzer, a platform for bacterial VF identification using the Virulence Factor Database-VFDB (https://www.mgc.ac.cn/VFs/ accessed on 16 January 2026) [11].

Genetic relatedness among the isolates was assessed using single nucleotide polymorphism (SNP)-based analysis with the CSI Phylogeny software (version 1.4), available on the CGE platform (https://cge.food.dtu.dk/services/CSIPhylogeny/ accessed on 16 January 2026 [12], with the *E. faecium* Aus0004 genome (Accession No. CP003351) as reference. The resulting SNP distance matrix was used to assess the genetic relationship among isolates, and an SNP difference cutoff < 20 was applied to define closely related isolates. The phylogenetic tree was visualized with Microreact (https://microreact.org/, accessed on 16 January 2026), developed by the Center for Genomic Pathogen Surveillance (CGPS) [13].

## 3. Results

### 3.1. Specimen Source for E. faecium VRE Isolates

A total of 44 *E. faecium* VRE isolates, stored by freeze preservation between 2021 and 2023, were selected based on total numbers of isolates per ward and year; forty-three of these isolates were from patients admitted to the Bolzano Regional Hospital, whereas one was from the Health District of Brunico.

Out of the examined isolates, 81.8% (36/44) were from clinical specimens of patients with suspected infections and 18.2% (8/44) were from rectal surveillance swabs (Table 1). Twenty-three patients (52.3%) were male and 21 (47.7%) were female. Patients’ mean age was 70.0 years (range 0–95 years), with 77.2% of isolates from patients over 60 years. Patients came from various hospital wards: Internal Medicine (No = 9), Intensive Care Unit (No = 7), General Surgery (No = 4), Gastroenterology (No = 4), Geriatrics Unit (No = 3), Neonatal Intensive Care Unit (No = 3), Vascular Surgery (No = 2), Hematology (No = 2), Urology (No = 2), Nephrology (No = 2), Casualty department (n = 1), Cardiology (No = 1), Dermatology (No = 1), Infectious Diseases Unit (No = 1), Gynecology (No = 1) and Otorhinolaryngology (No = 1).

### 3.2. Identification and Antimicrobial Susceptibility Testing Results

Isolates were identified by MALDI-TOF mass spectrometry, with scores > 2.0 (2.2 ± 0.1). Phenotypic susceptibility testing results are listed in Table 1. Resistance to vancomycin and teicoplanin (MICs > 16 mg/L, respectively) was found in all isolates except one, indicating a VanA phenotype; the exception was isolate No. 4424 with resistance to vancomycin and susceptibility to teicoplanin (MIC ≤ 0.5 mg/L), consistent with a VanB phenotype. For daptomycin, the MICs of all but one isolate (No. 5255) were ≤ the ECOFF value; for isolate No. 5255, the Vitek 2 test was also performed using the evaluation method for *E. faecalis*, which gave an MIC of 32 mg/L (Vitek 2 does not elaborate daptomycin MICs for *E. faecium*). Isolates No. 4497 and 5321 tested resistant to linezolid (>16 mg/L and 16 mg/L, respectively), whereas MICs for tedizolid were 0.5 mg/L and 2 mg/L, respectively (EUCAST clinical breakpoint not defined, ECOFF = 1 mg/L); for linezolid, Sensititre results were confirmed by Vitek 2. All isolates except one (No. 4750) were susceptible at a standard dosage regimen to quinupristin/dalfopristin, tigecycline and eravacycline. Oritavancin MICs for all isolates were ≤0.25 mg/L, but no EUCAST clinical breakpoints or ECOFF values have yet been defined. All MICs for dalbavancin except one (isolate No. 4424) were >2 mg/L, with Sensititre results confirmed by E-test. All isolates except two showed high-level resistance to gentamicin and streptomycin: isolate No. 5353 was high-level gentamicin susceptible, and isolate No. 4424 was high-level susceptible to gentamicin and streptomycin. All VREfm isolates except one (No. 4772) were resistant to the following antibiotics: ampicillin, ampicillin/sulbactam and amoxicillin/clavulanic acid (MICs >16 mg/L, Vitek 2), and had MICs >8 mg/L (Vitek 2) for imipenem and >8 mg/L for ceftaroline (Sensititre); isolate No. 4772, obtained in 2022 from the urine of a patient admitted to the Internal Medicine ward, was susceptible at a standard dosage regimen to ampicillin, ampicillin/sulbactam and amoxicillin/clavulanic acid (MICs ≤ 2 mg/L) and had an MIC ≤ 1 mg/L for imipenem and 0.06 mg/L for ceftaroline. Moreover, all isolates except one (No. 4615) were resistant to ciprofloxacin and levofloxacin (MICs > 4 mg/L, Vitek 2); the exception was isolate No. 4615, susceptible at a standard dosage regimen to ciprofloxacin (MIC ≤ 0.5 mg/L, Vitek 2) and levofloxacin (MIC = 2 mg/L, Vitek 2). All isolates except No. 4424 (MIC = 8 mg/L) had MICs > 32 mg/L for rifampicin (E-test). All isolates except No. 5353 and 4424 had MICs > 1 mg/L for clindamycin and >4 mg/L for erythromycin (Sensititre); MICs of isolates No. 5353 and 4424 for clindamycin and erythromycin were ≤0.125 mg/L.

### 3.3. Genomic Analysis

#### 3.3.1. Antimicrobial Resistance Genes

Resistome analysis using ResFinder is shown in Table 2. The *vanHAX* gene cluster, determining the VanA resistance phenotype, was found in all *E. faecium* isolates except No. 4424; the latter isolate had the *vanHBX* cluster, determining the VanB phenotype (Table 2). Therefore, the phenotype and genotype agreed for glycopeptides.

Aminoglycoside modifying resistance genes were the following: intrinsic *aac(6′)-Ii* (all isolates), acquired *aph(3′)-III* (42/44 isolates), *ant(6)-la* (39/44 isolates) and *aac(6)-aph(2″)* (9/44 isolates). A single isolate (No. 4424) was phenotypically susceptible to high dosages of gentamicin and streptomycin, confirmed by the only presence of the intrinsic *aac(6′)-Ii* aminoglycoside resistance gene (if present alone it does not cause HLR to gentamicin and streptomycin), whereas all other isolates had a combination of this intrinsic aminoglycoside resistance gene with other aminoglycoside modifying genes, which, together with reduced intrinsic susceptibility and possibly other resistance mechanisms, could explain the HLR phenotype for gentamicin and streptomycin.

Other resistance genes were *msr(C)* (42/44 isolates) coding for a macrolide efflux pump, the 28S rRNA modification genes *erm(B)* (41/44 isolates) and *erm(T)* (12/44 isolates) and the lincosamide modification gene *Inu(B)* together with the macrolide-streptogramin efflux pump gene *Isa(E)* (1/44 isolate), conferring resistance to macrolides, lincosamides and group B streptogramins (MLSB phenotype) in 42/44 isolates. Low MICs for clindamycin and erythromycin of the isolates No. 5353 and 4424 could be explained by the absence of *erm* genes (presence only of the *msr(C)* gene).

Another frequent resistance gene was *tet(L)* (27/44 isolates) coding for an efflux pump conferring resistance to tetracyclines, but tetracycline resistance was not tested (intrinsic resistance of *E. faecium*). Isolate No. 4424 contained the trimethoprim resistance gene *dfrG*, but we did not test isolates for resistance to this antibiotic.

Chromosomal point mutations were observed in the quinolone resistance determining regions (QRDRs) of the DNA gyrase (*gyrA*, S83I) (43/44 isolates) and DNA topoisomerase IV (*parC*, S80R) (41/44 isolates) genes; all of these 43 isolates were resistant to ciprofloxacin and levofloxacin. The exception was isolate No. 4615, susceptible at a standard dosage regimen to ciprofloxacin and levofloxacin; this isolate had no *gyrA* or *parC* mutation in the QRDRs.

LRE-Finder software showed the presence of a mutation in domain V of the gene for 23S rRNA, specifically G2576T, responsible for a linezolid resistance in isolate No. 4497, and of the transferable linezolid resistance genes *cfr(D)* and *optrA*, coding for a 23S rRNA methyltransferase and a ribosomal protection protein, respectively, in isolate No. 5321 [14].

Isolate No. 5255 showed a daptomycin MIC > 256 mg/L. By AMRFinderPlus, no point mutations in the *liaF*, *liaS* and *liaR* genes, associated with daptomycin resistance [15], were found in this isolate. Other resistance mechanisms were not further investigated.

#### 3.3.2. Multi Locus Sequence Typing (MLST)

Thirty-eight of the forty-four sequenced isolates belonged to ST80, 1 (No. 4424) to ST17 and 1 (No. 4615) to ST697 (Table 2). STs of 2 isolates (No. 4468 and No. 4861) are single locus variants (SLVs) of ST80, as they differ in only one of the seven canonical MLST typing loci; isolate No. 4791 differs in two alleles (double locus variant, DLV), whereas isolate No. 4809 differs by three alleles (tri locus variant, TLV).

#### 3.3.3. Virulence Factors

**Figure 1:**
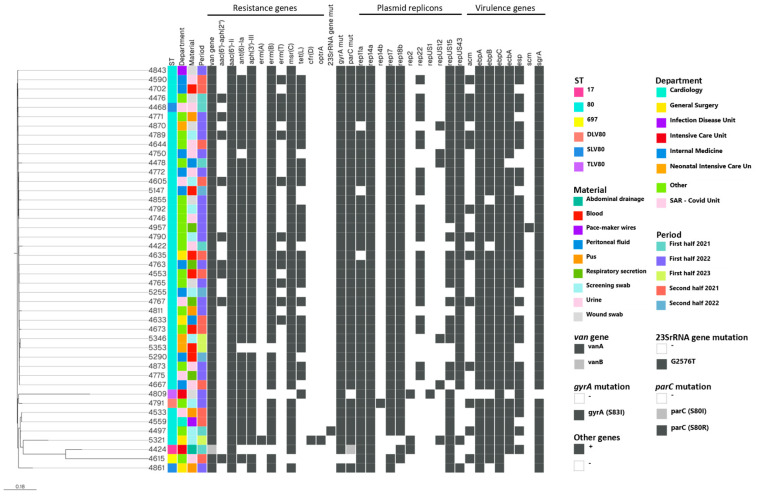
SNP-based core genome phylogeny of 44 VRE *E. faecium* isolates collected in the hospital of Bolzano (period 2021–2023). The phylogenetic tree was visualized by Microreact. Isolate names, clinical data and genome characteristics including resistance genes, plasmid replicons and virulence genes are at the right side of the tree. The legend includes the color-key used for each feature. SLV80, single locus variant of ST80; DLV80, dual locus variant of ST80; TLV80, tri locus variant of ST80; mut, mutation.

Analysis of the 44 VREfm genomes using VFDB revealed the presence of a total of 14 different virulence factor genes (Table S1, Figure 1). Most of the isolates harbored *sgrA* (43/44), *ecbA* (40/44) and *esp* (31/44), whereas *acm* was detected in 14 out of 44 isolates and *scm* in only one isolate (Figure 1). In addition, more than 90% of the isolates carried the pili gene cluster *ebpA-ebpB-ebpC*, which is involved in pilus formation. All of these genes encode surface proteins commonly associated with clinical isolates [16]. No significant differences in VF profiles were observed among the VREfm under study.

#### 3.3.4. Plasmids

Eleven different plasmid replicons in thirteen different combinations were identified in the 44 VREfm: *rep14a* in all isolates; *rep17* and *rep18b* each detected in 42 isolates; *rep11a* (n = 41); *repUS43* and *repUS15* each in 37 isolates; *rep22* (n = 23), *repUS12* (n = 6); *rep2* (n = 3); *rep14b* and *repUS1* each detected in only 1 isolate (Figure 1).

#### 3.3.5. Phylogenetic Analysis

Phylogenetic analysis based on core genome SNP differences showed that the majority (35) of the VREfm isolates clustered within a single group (Cluster 1), whereas the remaining 9 isolates appeared genetically distinct, with 2 of these forming a separate group (Cluster 2). Cluster 1 comprised closely related isolates showing pairwise SNP differences below 20, except for a few isolates with SNP differences from twenty-one to sixty-one (Figure 1), and in total belonging to ST80 (34) and to a SLV of ST80 (1) (Figure 1). Isolates showing 1–2 pairwise SNP differences were obtained either from the same or distinct hospital wards on closely or distantly spaced days. Cluster 2 consisted of two closely related isolates (No. 4533 and No. 4559) with six SNP differences, both belonging to ST80 and isolated in the second half of 2021 in two different wards at a twenty-day interval. The seven remaining isolates were identified as singletons as SNP differences ranged from 252 to 1070, indicating considerable genetic distance among them. They included the following: two isolates, No. 4497 and No. 5321 both belonging to ST80, that exhibited linezolid resistance associated with mutation in a 23S rRNA gene (No. 4497) and the presence of *cfr(D)* and *optrA* genes (No. 5321); No. 4424 (ST17) carrying *VanHBX*; No. 4615 (ST697); No. 4791 (DLV of ST80); No. 4809 (TLV of ST80); and isolate No. 4861 (SLV of ST80).

## 4. Discussion

In the Autonomous Province of Bolzano, Northern Italy, a significant and gradual increase in clinical isolates of *E. faecium* VRE has been registered in recent years, consistent with national and European data [4,5]. During 2019–2023, a total of 217 non-duplicate clinical isolates of VREfm were collected in the Bolzano Health District from all clinical sample types and settings (hospital isolates, nursing homes, outpatients), excluding surveillance culture isolates. Between 2019 and 2022, we registered a significant upward trend, followed by a trend reversal in 2023. In 2023, the percentage of patients with positive fecal surveillance cultures for VREfm widely varied among departments in the Bolzano Regional Hospital, with an average of 8.1% (unpublished data). A point prevalence study in a Bolzano Long-Term Care Facility involving both residents and staff of the facility, and a comparison with the colonization of patients admitted to the Geriatrics ward of the referral Bolzano Regional Hospital, showed a significant increase in colonization by VREfm in 2022 compared to 2008, 2012 and 2016 [17]. The high percentages of VREfm found in the Province of Bolzano are not in line with the general epidemiology of multi-drug resistant (MDR) bacteria in the same geographical area, as MDR bacteria isolated from blood/liquor are generally significantly less prevalent than the Italian averages. In general, the Province of Bolzano records the lowest antibiotic resistance prevalences of all Italian provinces/regions and these are also generally below the respective European averages [5].

In the present study, 44 VREfm isolates collected during 2021–2023 were phenotypically and molecularly characterized for the first time in the Province of Bolzano. The tested isolates were highly multiresistant but generally maintained susceptibility and/or low MICs for daptomycin, linezolid, quinupristin/dalfopristin, oritavancin, tigecycline and eravacycline.

All isolates except one had the VanA phenotype, and genomic analysis showed the presence of the *vanHAX* gene cluster; the single isolate with a VanB phenotype was obtained in 2021 from a patient admitted to the Intensive Care Unit, and genomic analysis confirmed the presence of the *vanHBX* gene cluster. Isolates of VanB type VREfm have so far only sporadically been found in the Province of Bolzano. AR-ISS data suggest a high prevalence of the VanA phenotype in Italy in 2023, with resistance rates for blood/liquor isolates of 32.5% for vancomycin and 31.9% for teicoplanin [5].

Oritavancin is the only lipoglycopeptide active against all VREs, regardless of the Van resistance phenotype; in contrast, telavancin and dalbavancin appear to be active only against VanB-type VREs [18,19]. Although an ECOFF value and a EUCAST breakpoint for oritavancin have not yet been established, all MICs of our isolates were ≤0.25 mg/L, indicating this antibiotic as a potential therapeutic option for infections caused by VREfm [20].

Genomic analysis of the 44 isolates showed that 38 of them (86.3%) belonged to ST80, 4 were single (SLVs), double (DLV) or triple (TLV) locus variants of ST80 (9.1%), one was ST17 and one was ST697. The ST80 and ST17 strains of *E. faecium* belong to the clonal complex CC17, which is characterized by vancomycin-resistance, the presence of various virulence determinants and biofilm formation [21]. The hospital-adapted CC17 lineage of *E. faecium*, responsible for urinary tract infections, endocarditis and other infection types, is associated with severe morbidity and mortality and causes a considerable economic burden on public health [22].

As shown for our study isolates, the nosocomial transmission of *E. faecium* is reflected in the high overall clonality rate, with only one dominant sequence type found. Compared to community-adapted *E. faecium* isolates, those adapted to the hospital environment and belonging to CC17 contain more exogenously acquired genetic material, such as pathogenicity islands (PAIs) and plasmids or other mobile genetic elements frequently associated with antimicrobial resistance, virulence and/or colonization and carbohydrate metabolism [23]. These peculiarities give *E. faecium* CC17 selective advantages, making it difficult to treat in hospital settings and in certain patient niches [21].

A 2019 Danish study [24] and a retrospective study conducted in an Italian regional hospital [25] demonstrated that *E. faecium* ST80 was the most prevalent lineage in their hospital settings. Moreover, a single ST697 strain, not belonging to the CC17, was isolated from a patient admitted to the Geriatrics Unit but resident in a nursing home in Bolzano. In Public databases for molecular typing and microbial genome diversity (PubMLST), a single strain of *E. faecium* ST697 is reported, isolated in Spain in 2010 from a stool sample collected from a hospitalized patient [26].

Regarding virulence factors, all of our isolates except ST697 had a similar genetic profile. Phylogenetic analysis allowed to establish the clonality of most of our ST80 isolates, and, in addition, epidemiological and phylogenetic data were found to correspond. The isolates were found spatially dispersed throughout the hospital wards during the period from January 2021 to April 2023, suggesting a widespread transmission.

Two VREfm isolates included in the study were resistant to linezolid, but the percentage of linezolid-resistant *E. faecium* isolates in the Province of Bolzano in 2022 and 2023 was ≤0.5% (unpublished data). The two linezolid-resistant *E. faecium* isolates had MICs for tedizolid of 0.5 and 2 mg/L (ECOFF = 1 mg/L). In one linezolid-resistant isolate, sequencing analyses revealed the presence of a G2576T point mutation in the loop of the V-domain of the ribosomal 23S rRNA, which is the mutation most frequently associated with linezolid resistance, although it was not possible to specify in how many of the six chromosomal genes for 23S rRNA this mutation was present [27]. Furthermore, in one of the isolates, the transferable linezolid resistance genes *cfr(D)* and *optrA* were found. Other authors have described the presence of the *cfr* and *optrA* genes on a plasmid from an Italian *E. faecium* blood isolate obtained in 2015 [28].

In general, the resistance genes found in our isolates are in line with the respective phenotypes. However, the lack of identification of some genes linked to antibiotic resistance and virulence factors does not allow their absence from the genome to be ruled out with certainty, because, due to procedural limitations, genomes are not ‘closed’ but are instead subdivided into numerous contigs. The fragmentation of the genome means that there may be gaps (ranging from a few to several hundred base pairs) in which part or all of the gene sequence may fall, preventing the identification of the relevant gene.

A limitation of our study is the characterization of a small percentage of total isolates (16.5% of all first clinical routine VREfm isolates and a lower percentage of surveillance isolates), obtained during the period 2021–2023 in the Bolzano Health District. A further limitation is that it was performed in a single health district in the Province of Bolzano (except for a single isolate from another health district in the same province), not allowing for the extrapolation of data to other health districts in the same province or other provinces/regions in Italy. Moreover, to consolidate the significance of the data obtained, the evaluation of information regarding the clinical status of patients and its correlation with virulence factors and antimicrobial resistance profiles is desirable, but the limited number of sequenced isolates did not allow for a similar analysis.

VREfm strains are emerging pathogens causing a significant number of healthcare-associated infections (HAIs) [29]. Based on these considerations, it is essential to take effective preventive infection control and antimicrobial stewardship measures against VRE infections/colonizations, especially in hospitalized patients. The increasing trend of VREfm infections/colonization in the Bolzano Regional Hospital during the period 2019–2022, followed by a decreasing trend in 2023, correlates temporally with the COVID-19 pandemic and therefore a causal relationship is possible [30]. The combined implementation of hygienic–sanitary interventions is crucial for reducing or eliminating VRE infections/colonizations in hospital departments, as demonstrated by the decreasing trend for VREfm observed during 2023 in the Bolzano Regional Hospital, when enhanced surveillance and infection control procedures according to a locally adapted guideline have been applied, based on the infection prevention guideline for VRE infections of the Robert Koch Institute in Germany [31].

## 5. Conclusions

The present study shows that it is essential to provide the molecular typing of the VREfm isolates in association with phenotypic characterizations. This combined approach allows a better understanding of the spread and evolution of VREfm strains in hospital settings, providing a solid basis for the development of infection control and prevention strategies. The results can also serve as a basis for future studies to determine the origin and routes of infection, as well as to confirm or exclude outbreaks, track the cross-transmission of healthcare-associated pathogens, recognize particularly virulent strains and evaluate the effectiveness of control measures in the context of the infection prevention and control. In addition to the application of up-to-date infection control strategies and antibiotic stewardship programs, future activities should focus on the implementation of appropriate surveillance systems, including genomic analyses.

## Figures and Tables

**Table 1:** Antimicrobial susceptibility testing results for 44 *E. faecium* VRE isolates and elaboration according to EUCAST breakpoints and/or ECOFF values.

Isolates	Materials	VAN (∗)	TPN (∗∗)	DAL (∗)	TLA (∗)	ORI (∗)	DAP (∗∗∗)	LZD (∗)	TZD (∗)	QDA (∗∗)	TGC (∗∗)	ERV (∗∗∗)	HLG (∗∗)	HLS (∗∗)	FOS (∗∗∗)	NIT (∗∗∗)
4422	Urine	>16 (R)	>16 (R)	>2	1	0.008	2 (S)	1(S)	0.25	0.5 (S)	≤0.125(S)	0.03 (S)	SYN-R	SYN-R	128	>512
4424	Abdominal drainage	>16 (R)	<0.5 (S)	0.03	0.016	0.001	1.5 (S)	2 (S)	0.25	<0.25 (S)	≤0.125 (S)	0.016 (S)	SYN-S	SYN-S	32	32
4468	Urine	>16 (R)	>16 (R)	>2	1	0.016	4 (S)	2 (S)	0.5	0.5 (S)	≤0.125 (S)	0.03 (S)	SYN-R	SYN-R	128	>512
4476	Wound swab	>16 (R)	>16 (R)	>2	2	0.016	4 (S)	2 (S)	0.5	0.5 (S)	≤0.125 (S)	0.03 (S)	SYN-R	SYN-R	>1024	>512
4478	Peritoneal fluid	>16 (R)	>16 (R)	>2	1	0.016	4 (S)	2 (S)	0.5	0.5 (S)	≤0.125 (S)	0.125 (S)	SYN-R	SYN-R	128	>512
4497	Rectal surveillance swab	>16 (R)	>16 (R)	>2	1	0.008	4 (S)	**>16 (R)**	0.5	0.5 (S)	≤0.125 (S)	0.016 (S)	SYN-R	SYN-R	128	>512
4533	Pus	>16 (R)	>16 (R)	>2	1	0.016	3 (S)	2 (S)	0.5	0.5 (S)	≤0.125 (S)	0.016 (S)	SYN-R	SYN-R	128	>512
4553	Blood	>16 (R)	>16 (R)	>2	1	0.008	3 (S)	1 (S)	0.125	0.5 (S)	≤0.125 (S)	0.06 (S)	SYN-R	SYN-R	128	>512
4559	Pace-maker wires	>16 (R)	>16 (R)	>2	2	0.016	3 (S)	2 (S)	0.5	0.5 (S)	≤0.125 (S)	0.012(S)	SYN-R	SYN-R	128	>512
4590	Urine	>16 (R)	>16 (R)	>2	2	0.03	4 (S)	2 (S)	0.5	0.5 (S)	≤0.125 (S)	0.03 (S)	SYN-R	SYN-R	128	>512
4605	Rectal surveillance swab	>16 (R)	32 (R)	>2	1	0.008	3 (S)	1 (S)	0.125	0.5 (S)	≤0.125 (S)	0.06 (S)	SYN-R	SYN-R	128	>512
4615	Urine	>16 (R)	>16 (R)	>2	1	0.008	3 (S)	2 (S)	0.5	0.5 (S)	≤0.125 (S)	0.03 (S)	SYN-R	SYN-R	128	64
4633	Peritoneal fluid	>16 (R)	>16 (R)	>2	2	0.016	3 (S)	2 (S)	0.5	0.5 (S)	≤0.125 (S)	0.125 (S)	SYN-R	SYN-R	192	>512
4635	Blood	>16 (R)	>16 (R)	>2	2	0.016	3 (S)	2 (S)	0.5	0.5 (S)	≤0.125 (S)	0.06 (S)	SYN-R	SYN-R	128	256
4644	Urine	>16 (R)	16 (R)	>2	2	0.016	3 (S)	2 (S)	0.5	0.5 (S)	≤0.125 (S)	0.06 (S)	SYN-R	SYN-R	>1024	>512
4667	Urine	>16 (R)	>16 (R)	>2	2	0.03	3 (S)	2 (S)	0.5	1 (S)	≤0.125 (S)	0.125 (S)	SYN-R	SYN-S	128	256
4673	Blood	>16 (R)	>16 (R)	>2	2	0.03	3 (S)	2 (S)	0.25	0.5 (S)	≤0.125 (S)	0.125 (S)	SYN-R	SYN-R	128	>512
4702	Blood	>16 (R)	>16 (R)	>2	2	0.016	3 (S)	2 (S)	0.5	0.5 (S)	≤0.125 (S)	0.06 (S)	SYN-R	SYN-R	128	256
4719	Rectal surveillance swab	>16 (R)	>16 (R)	>2	2	0.016	3 (S)	2 (S)	0.5	0.5 (S)	≤0.125 (S)	0.06 (S)	SYN-R	SYN-R	128	256
4746	Urine	>16 (R)	>16 (R)	>2	2	0.016	4 (S)	2 (S)	0.5	0.5 (S)	≤0.125 (S)	0.125 (S)	SYN-R	SYN-R	256	256
4750	Urine	>16 (R)	>16 (R)	>2	2	0.016	2 (S)	2 (S)	0.5	**2 (R)**	≤0.125 (S)	0.06 (S)	SYN-R	SYN-R	128	256
4763	Respiratory secretion	>16 (R)	>16 (R)	>2	>2	0.016	3 (S)	2 (S)	0.5	0.5 (S)	≤0.125 (S)	0.06 (S)	SYN-R	SYN-R	128	>512
4765	Ulcer swab	>16 (R)	>16 (R)	>2	2	0.03	4 (S)	2 (S)	0.5	1 (S)	≤0.125 (S)	0.06 (S)	SYN-R	SYN-R	128	>512
4767	Respiratory secretion	>16 (R)	>16 (R)	>2	2	0.016	3 (S)	2 (S)	0.5	0.5 (S)	≤0.125 (S)	0.06 (S)	SYN-R	SYN-R	128	>512
4771	Pus	>16 (R)	>16 (R)	>2	2	0.016	3 (S)	2 (S)	0.5	0.5 (S)	≤0.125 (S)	0.032 (S)	SYN-R	SYN-R	128	256
4772	Urine	>16 (R)	>16 (R)	>2	2	0.016	3 (S)	2 (S)	0.5	0.5 (S)	≤0.125 (S)	0.06 (S)	SYN-R	SYN-R	64	256
4775	Respiratory secretion	>16 (R)	>16 (R)	>2	2	0.016	3 (S)	2 (S)	0.5	0.5 (S)	≤0.125 (S)	0.06 (S)	SYN-R	SYN-R	128	>512
4789	Rectal surveillance swab	>16 (R)	>16 (R)	>2	2	0.03	3 (S)	2 (S)	0.5	0.5 (S)	≤0.125 (S)	0.125 (S)	SYN-R	SYN-R	128	>512
4790	Rectal surveillance swab	>16 (R)	>16 (R)	>2	2	0.03	3 (S)	2 (S)	0.5	0.5 (S)	≤0.125 (S)	0.125 (S)	SYN-R	SYN-R	128	256
4791	Rectal surveillance swab	>16 (R)	>16 (R)	>2	2	0.03	4 (S)	2 (S)	0.5	0.5 (S)	≤0.125 (S)	0.06 (S)	SYN-R	SYN-R	128	>512
4792	Rectal surveillance swab	>16 (R)	>16 (R)	>2	2	0.03	2(S)	2 (S)	0.5	0.5 (S)	≤0.125 (S)	0.125 (S)	SYN-R	SYN-R	128	256
4809	Wound swab	>16 (R)	>16 (R)	>2	2	0.03	4 (S)	2 (S)	0.5	1 (S)	≤0.125 (S)	0.125 (S)	SYN-R	SYN-R	128	256
4811	Pus	>16 (R)	>16 (R)	>2	>2	0.25	4 (S)	4 (S)	0.5	0.5 (S)	≤0.125 (S)	0.06 (S)	SYN-R	SYN-R	128	>512
4843	Wound swab	>16 (R)	>16 (R)	>2	2	0.008	1(S)	2 (S)	0.5	0.5 (S)	≤0.125 (S)	0.06 (S)	SYN-R	SYN-R	32	256
4855	Ulcer swab	>16 (R)	>16 (R)	>2	2	0.03	2(S)	2 (S)	0.5	0.5 (S)	≤0.125 (S)	0.06 (S)	SYN-R	SYN-R	128	256
4861	Pus	>16 (R)	>16 (R)	>2	2	0.03	2(S)	2 (S)	0.5	0.5 (S)	≤0.125 (S)	0.03 (S)	SYN-R	SYN-R	128	>512
4870	Wound swab	>16 (R)	>16 (R)	>2	2	0.03	2(S)	2 (S)	0.5	0.5 (S)	≤0.125 (S)	0.06 (S)	SYN-R	SYN-R	128	256
4873	Urine	>16 (R)	>16 (R)	>2	2	0.06	4 (S)	2 (S)	0.5	0.5 (S)	≤0.125 (S)	0.125 (S)	SYN-R	SYN-R	128	256
4957	Respiratory secretion	>16 (R)	>16 (R)	>2	2	0.03	3 (S)	2 (S)	0.5	0.5 (S)	≤0.125 (S)	0.125 (S)	SYN-R	SYN-S	128	256
5018	Respiratory secretion	>16 (R)	>16 (R)	>2	>2	0.03	4 (S)	4 (S)	0.5	0.5 (S)	≤0.125 (S)	0.06 (S)	SYN-R	SYN-R	128	>512
5033	Urine	>16 (R)	>16 (R)	>2	2	0.016	3 (S)	2 (S)	0.25	0.5 (S)	≤0.125 (S)	0.06 (S)	SYN-R	SYN-R	128	256
5147	Blood	>16 (R)	>16 (R)	>2	2	0.016	3 (S)	2 (S)	0.5	0.5 (S)	≤0.125 (S)	0.03 (S)	SYN-R	SYN-R	128	256
5255	Rectal surveillance swab	>16 (R)	>16 (R)	>2	2	0.016	**>256 (R)**	2 (S)	0.5	0.5 (S)	≤0.125 (S)	0.03 (S)	SYN-R	SYN-R	128	32
5290	Blood	>16 (R)	>16 (R)	>2	2	0.016	3 (S)	2 (S)	0.5	0.5 (S)	≤0.125 (S)	0.03 (S)	SYN-R	SYN-R	128	256
5321	Rectal surveillance swab	>16 (R)	>16 (R)	>2	>2	0.016	3 (S)	**16 (R)**	2	0.5 (S)	≤0.125 (S)	0.03 (S)	SYN-R	SYN-R	64	64
5346	Rectal surveillance swab	>16 (R)	>16 (R)	>2	2	0.03	2(S)	2 (S)	0.5	0.5 (S)	≤0.125 (S)	0.125 (S)	SYN-R	SYN-R	128	256
5353	Blood	>16 (R)	>16 (R)	>2	2	0.016	4 (S)	2 (S)	0.5	0.5 (S)	≤0.125 (S)	0.03 (S)	SYN-S	SYN-R	128	>512
ECOFF (mg/L), EUCAST		4	2	/	0.5	/	8	4	1	2	0.25	0.125	/	/	128	256
% >ECOFF		100.0%	97.9%	/	97.9%	/	2.1%	4.3%	2.1%	0.0%	0.0%	0.0%	/	/	6.4%	91.5%
Breakpoint S (mg/L), EUCAST		≤4	≤2	/	/	/	/	≤4	/	≤1	≤0.25	0.125	/	/	/	/
Breakpoint R (mg/L), EUCAST		>4	>2	/	/	/	/	>4	/	>1	>0.25	0.125	/	/	/	/
% R		100.0%	97.9%	/	/	/	2.1%	4.3%	/	0.0%	0.0%	0.0%	95.7%	95.7%	/	/

Susceptibility testing method: ∗ Vitek 2, ∗∗ Sensititre, ∗∗∗ E-test. Antibiotics: VAN = vancomycin, TPN = teicoplanin, DAL = dalbavancin, TLA = telavancin, ORI = oritavancin, DAP = daptomycin, LZD = linezolid, TZD = tedizolid, QDA = quinupristin/dalfopristin, TGC = tigecycline, ERV = eravacycline, HLG = gentamicin high-level resistance, HLS = streptomycin high-level resistance, SYN-S = synergy between aminoglycoside and beta-lactam antibiotic or glycopeptide, SYN-R = no synergy between aminoglycoside and beta-lactam antibiotic or glycopeptide, FOS = fosfomycin, NIT = nitrofurantoin. MICs in mg/L. EUCAST interpretation in brackets (S: susceptible, standard dosing regimen; R: resistant).

**Table 2:** Sequence types (STs), antibiotic resistance genes and mutations, and plasmid replicons for 44 *E. faecium* VRE isolates.

Isolate Number	ST	Resistance Genes
4346, 4478, 4644, 4673, 4702, 4746, 4772, 4957, 4855, 4559, 4775, 4792, 4811, 4870, 4873, 5255, 5290	80	*aac(6′)-Ii*, *ant(6)-Ia*, *aph(3′)-III*, *erm(B)*, *gyrA (S83I)*, *msr(C)*, *parC (S80R)*, *tet(L)*, *vanHAX*
4422, 4533, 4559, 4811	80	*aac(6′)-Ii*, *ant(6)-Ia*, *aph(3′)-III*, *erm(B)*, *gyrA (S83I)*, *msr(C)*, *parC (S80R)*, *vanHAX*
4476, 4771	80	*aac(6′)-aph(2″)*, *aac(6′)-Ii*, *ant(6)-Ia*, *aph(3′)-III*, *erm(B)*, *erm(T)*, *gyrA (S83I)*, *msr(C)*, *parC (S80R)*, *tet(L)*, *vanHAX*
4590, 4633	80	*aac(6′)-Ii*, *ant(6)-Ia*, *aph(3′)-III*, *erm(B)*, *erm(T)*, *gyrA (S83I)*, *msr(C)*, *parC (S80R)*, *tet(L)*, *vanHAX*
4750, 4843	80	*aac(6′)-Ii*, *aph(3′)-III*, *erm(B)*, *gyrA (S83I)*, *msr(C)*, *parC (S80R)*, *vanHAX*
4767, 4789	80	*aac(6′)-aph(2″)*, *aac(6′)-Ii*, *ant(6)-Ia*, *aph(3′)-III*, *erm(B)*, *erm(T)*, *gyrA (S83I)*, *msr(C)*, *parC (S80R)*, *tet(L)*, *vanHAX*
4424	17	*aac(6′)-Ii*, *dfrG*, *gyrA (S83I)*, *msr(C)*, *parC (S80I)*, *vanHBX*
4468	SLV80	*aac(6′)-Ii*, *ant(6)-Ia*, *aph(3′)-III*, *erm(B)*, *erm(T)*, *gyrA (S83I)*, *msr(C)*, *parC (S80R)*, *tet(L)*, *vanHAX*
4497	80	*aac(6′)-Ii*, *ant(6)-Ia*, *aph(3′)-III*, *erm(B)*, *gyrA (S83I)*, *msr(C)*, *parC (S80R)*, *G2576U 23S rRNA*, *vanHAX*
4553	80	*aac(6′)-aph(2″)*, *aac(6′)-Ii*, *ant(6)-Ia*, *aph(3′)-III*, *erm(B)*, *gyrA (S83I)*, *msr(C)*, *parC (S80R)*, *tet(L)*, *vanHAX*
4605	80	*aac(6′)-aph(2′’)*, *aac(6′)-Ii*, *ant(6)-Ia*, *aph(3′)-III*, *erm(B)*, *erm(T)*, *gyrA (S83I)*, *msr(C)*, *parC (S80R)*, *tet(L)*, *vanHAX*
4615	697	*aac(6′)-aph(2″)*, *aac(6′)-Ii*, *ant(6)-Ia*, *aph(3′)-III*, *erm(B)*, *vanHAX*
4635	80	*aac(6′)-Ii*, *ant(6)-Ia*, *aph(3′)-III*, *erm(B)*, *erm(T)*, *gyrA (S83I)*, *msr(C)*, *parC (S80R)*, *tet(L)*, *vanHAX*
4763	80	*aac(6′)-aph(2″)*, *aac(6′)-Ii*, *ant(6)-Ia*, *aph(3′)-III*, *erm(B)*, *erm(T)*, *gyrA (S83I)*, *msr(C)*, *parC (S80R)*, *tet(L)*, *vanHAX*
4765	80	*aac(6′)-Ii*, *ant(6)-Ia*, *aph(3′)-III*, *erm(B)*, *erm(T)*, *gyrA (S83I)*, *msr(C)*, *parC (S80R)*, *tet(L)*, *vanHAX*
4790	80	*aac(6′)-aph(2″)*, *aac(6′)-Ii*, *ant(6)-Ia*, *aph(3′)-III*, *erm(B)*, *erm(T)*, *gyrA (S83I)*, *msr(C)*, *parC (S80R)*, *tet(L)*, *vanHAX*
4791	DLV80	*aac(6′)-Ii*, *ant(6)-Ia*, *aph(3′)-III*, *erm(B)*, *gyrA (S83I)*, *msr(C)*, *parC (S80R)*, *vanHAX*
4809	TLV80	*aac(6′)-Ii*, *ant(6)-Ia*, *aph(3′)-III*, *gyrA (S83I)*, *lnu((B)*, *Isa(E)*, *tet(L)*, *vanHAX*
4861	SLV80	*aac(6′)-Ii*, *aph(3′)-III*, *erm(B)*, *gyrA (S83I)*, *msr(C)*, *parC (S80R)*, *vanHAX*
5321	80	*aac(6′)-Ii*, *ant(6)-Ia*, *aph(3′)-III*, *cfr(D)*, *erm(A)*, *erm(B)*, *gyrA (S83I)*, *msr(C)*, *optr(A)*, *parC (S80R)*, *vanHAX*
5353	80	*aac(6′)-Ii*, *gyrA (S83I)*, *msr(C)*, *parC (S80R)*, *tet(L)*, *vanHAX*

## Data Availability

All genome sequences in this study were submitted to NCBI under the project accession number PRJNA1227293.

## Supplementary Materials

The following supporting information can be downloaded at https://www.mdpi.com/article/10.3390/pathogens15020143/s1, Table S1: Analysis of virulence factor genes in 44 VREfm isolates.

## Abbreviations

The following abbreviations are used in this manuscript:

DLV	Double Locus Variant
DNA	Deoxyribonucleic Acid
ECOFF	EUCAST Epidemiological Cut-Off Value
EUCAST	European Committee on Antimicrobial Susceptibility Testing
HLR	High-Level Resistance
MALDI-TOF	Matrix-Assisted Laser Desorption/Ionization-Time of Flight
MDR	Multi-Drug Resistant
MIC	Minimal Inhibitory Concentration
MLST	Multi Locus Sequencing Type
QRDR	Quinolone Resistance Determining Region
RNA	Ribonucleic Acid
SLV	Single Locus Variant
SNPs	Single Nucleotide Polymorphisms
ST	Sequence Type
TLV	Tri Locus Variant
VF	Virulence Factor
VRE	Vancomycin-Resistant Enterococci
VREfm	Vancomycin-Resistant *Enterococcus faecium*
WGS	Whole Genome Sequencing
